# Gene Electrotransfer of Plasmid with Tissue Specific Promoter Encoding shRNA against Endoglin Exerts Antitumor Efficacy against Murine TS/A Tumors by Vascular Targeted Effects

**DOI:** 10.1371/journal.pone.0124913

**Published:** 2015-04-24

**Authors:** Monika Stimac, Tanja Dolinsek, Ursa Lampreht, Maja Cemazar, Gregor Sersa

**Affiliations:** 1 Department of Experimental Oncology, Institute of Oncology Ljubljana, Ljubljana, Slovenia; 2 Faculty of Health Sciences, University of Primorska, Izola, Slovenia; Swedish Neuroscience Institute, UNITED STATES

## Abstract

Vascular targeted therapies, targeting specific endothelial cell markers, are promising approaches for the treatment of cancer. One of the targets is endoglin, transforming growth factor-β (TGF-β) co-receptor, which mediates proliferation, differentiation and migration of endothelial cells forming neovasculature. However, its specific, safe and long-lasting targeting remains the challenge. Therefore, in our study we evaluated the transfection efficacy, vascular targeted effects and therapeutic potential of the plasmid silencing endoglin with the tissue specific promoter, specific for endothelial cells marker endothelin-1 (ET) (TS plasmid), in comparison to the plasmid with constitutive promoter (CON plasmid), *in vitro* and *in vivo*. Tissue specificity of TS plasmid was demonstrated *in vitro* on several cell lines, and its antiangiogenic efficacy was demonstrated by reducing tube formation of 2H11 endothelial cells. *In vivo*, on a murine mammary TS/A tumor model, we demonstrated good antitumor effect of gene electrotransfer (GET) of either of both plasmids in treatment of smaller tumors still in avascular phase of growth, as well as on bigger tumors, already well vascularized. In support to the observations on predominantly vascular targeted effects of endoglin, histological analysis has demonstrated an increase in necrosis and a decrease in the number of blood vessels in therapeutic groups. A significant antitumor effect was observed in tumors in avascular and vascular phase of growth, possibly due to both, the antiangiogenic and the vascular disrupting effect. Furthermore, the study indicates on the potential use of TS plasmid in cancer gene therapy since the same efficacy as of CON plasmid was determined.

## Introduction

Antiangiogenic therapies are promising approaches for the treatment of cancer [1]. Forty years ago, the study of Folkman *et al*. [2], was the first associating tumor growth with angiogenesis, followed by many studies demonstrating that tumors depend on angiogenesis to grow beyond few mm^3^ in order to provide oxygen and nutrients [3,4]. Tumor vasculature consists of fast proliferating endothelial cells that have specific endothelial cell markers, which can be targeted by several antiangiogenic therapies. The most studied cell markers are vascular endothelial growth factor (VEGF) and its receptors [5,6]. However, due to the severe side effects of VEGF based therapies and in the search for potential use in the combined therapeutic approach with other vascular targeted approaches, new targets are being sought. One of them is endoglin (CD105), a transforming growth factor β (TGF-β) co-receptor, involved in cellular proliferation, differentiation and migration [7,8]. Its expression is highly elevated in endothelial cells within tumors and in the tumor surrounding blood vessels [9,10]. Therefore, targeting endoglin has been explored as a therapeutic option in preclinical [11–13] and clinical setting [14–16].

Different approaches can be used to target endoglin, such as differently conjugated antibodies, small interfering RNAs (siRNA) or plasmids. Studies *in vitro* have demonstrated that anti-endoglin nanobodies [17] and different antibodies inhibit proliferation, migration and adhesion of endothelial cells [14,18]. This led to several studies *in vivo*, using different anti-endoglin antibodies; naked [19–21], immunotoxin-conjugated [22–24] or radiolabeled [25], which demonstrated good antitumor response. Furthermore, by using gene electrotransfer (GET) of siRNA molecules *in vitro* and *in vivo*, we demonstrated pronounced antitumor and specific antiangiogenic response [12].

The first demonstration of tumor regression when using GET of plasmid encoding Stat-3, was published by Niu *et al*. in 1999 [26], followed by many other studies targeting different molecules such as HIV-1 Vpr [27], Plk 1 and Bcl-2 [28], VEGF [5], integrins [29] and also others stimulating the immune response, for example IL-12 [30–32], IL-15 [33], IFNα [34] alone or in combination with other therapies, as for example chemotherapy and radiotherapy [35,36]. Those preclinical studies have led to the fist clinical trials using GET of plasmid encoding human IL-12 (hIL-12) [37] and plasmid AMEP for binding on integrins for treatment of melanoma [38].

In our recent study, we were the first to demonstrate that silencing endoglin with GET, using plasmid with constitutive promoter (CON plasmid), has pronounced antitumor efficacy in murine tumor model [11]. In order to specifically silence endoglin within tumor vasculature, plasmid with tissue specific promoter represents good option. We used tissue specific promoter, which is endothelin-1 (ET) dependent and it is involved in migration of endothelial cells (TS plasmid). With such approach, one can achieve higher specificity, safety and yet the same efficacy as using CON plasmid. With the aim to evaluate its transfection efficacy and vascular targeted effect, in our study, we performed serial tests *in vitro* and *in vivo*. *In vitro*, we confirmed the tissue specificity of TS plasmid and its antiangiogenic potential on tube formation of endothelial cells. To further evaluate vascular targeted and antitumor effects *in vivo*, we used murine mammary carcinoma TS/A, in which the tumor cells do not express endoglin [12] and ET [39]. Since the tumor size can have an impact on the outcome of the different therapies [40], in our study the same therapeutic protocol was performed on tumors of different size and thus different vascularity. We observed that antitumor effect was more pronounced on smaller tumors in avascular phase of growth, rather than on bigger tumors with highly developed tumor blood vessels. Histological analysis demonstrated a significant increase in necrosis and decrease in the number of tumor blood vessels. In addition, the effect was the same when using either of both plasmids silencing endoglin; CON and TS plasmid, indicating on the therapeutic potential of the latter.

## Materials and Methods

### Cell lines

Murine mammary adenocarcinoma cells TS/A [41] and murine melanoma cell lines; B16F1 and B16F10 (American Type Culture Collection (ATCC), Manassas, VA, USA) were cultured in advanced minimum essential medium (AMEM, Life Technologies, Grand Island, NY, USA) supplemented with 5% fetal bovine serum (FBS, Life Technologies), 10 mM/l L-glutamine (Life Technologies), 100 U/ml penicillin (Grünenthal, Aachen, Germany) and 50 mg/ml gentamicin (Krka, Novo Mesto, Slovenia) in a 5% CO_2_ humidified incubator at 37°C.

Murine endothelial cell line 2H11 (ATCC) and human umbilical vein endothelial cell line HUVEC (HUV-EC-C cells that are commercially available from ATCC (HUV-EC-C (ATCC CRL-1730) were cultured in advanced Dulbecco modified Eagle medium (DMEM, Life Technologies) supplemented with 5% FBS, 10 mM/l L-glutamine, 100 U/ml penicillin and 50 mg/ml gentamicin in a 5% CO_2_ humidified incubator at 37°C.

Human microvascular endothelial cell line HMEC-1 (Centers for Disease Control and Prevention, Atlanta, GA, USA) was cultured in MCDB 131 medium (Life Technologies) supplemented with 10% FBS, 10 mM/l L-glutamine, 10 μg/l epidermal growth factor (Life Technologies), 1 mg/l hydrocortisone (Sigma Aldrich, St. Louis, MO, USA), 100 U/ml penicillin and 50 mg/ml gentamicin in a 5% CO_2_ humidified incubator at 37°C.

### Plasmids

Two different plasmids encoding enhanced green fluorescent protein (EGFP) were used for obtaining flow cytometry data *in vitro*; pET-EGFP [42] and pEGFP-N1 (Clontech, Basingstoke, UK). pET-EGFP, recombinant plasmid expressing EGFP under the control of tissue specific polymerase II promoter for endothelial cell marker endothelin-1 (ET) [42], was used for determination of plasmids`tissue specificity for endothelial cells on different cell lines after GET. For comparison, pEGFP-N1 encoding EGFP under the control of CMV polymerase II constitutive promoter was used.

Two plasmids silencing endoglin by encoding shRNA against it were used in further experiments; one with tissue specific polymerase II promoter for endothelial cell marker ET (pET-antiCD105; TS plasmid) and one with polymerase III U6 constitutive promoter (pU6-antiCD105; CON plasmid) [11]. A plasmid encoding shRNA with no homology to any gene in the mouse genome under the control of polymerase III U6 constitutive promoter was used as negative control (pU6-SCR; SCR plasmid) [11].

All plasmids were isolated using JETSTAR 2.0 ENDOTOXIN-FREE Plasmid MEGA Kit (Genomed, Löhne, Germany) and diluted in endotoxin free water to a concentration of 1 μg/μl (*in vitro* experiments) and 4 μg/μl (*in vivo* experiments). Concentrations of plasmids were measured with a spectrophotometer at 260 nm (Epoch Microplate Spectrophotometer, Take3 Micro-Volume Plate, BioTek, Bad Friedrichshall, Germany) and purity of plasmid was determined by agarose gel electrophoresis and measurements of the absorbance ratio at 260 and 280 nm.

### *In vitro* GET

A monolayer of 80% confluent cell cultures was trypsinized, washed with corresponding supplemented medium and collected in a cell suspension in ice-cold electroporation buffer (EP buffer: 125 mM sucrose, 10 mM K_2_HPO_4_, 2.5 mM KH_2_PO_4_, 2 mM MgCl_2_ x 6H_2_O). For electroporation, the cell suspension was prepared in ice-cold EP buffer (25 x 10^6^ cells/ml) and was later divided into several aliquots of 44 μl. In each aliquot, 11 μl of either of the plasmids (1 μg/μl) or endotoxin-free water were added. From the mixture, 50 μl were pipetted between two stainless steel parallel plate electrodes with a 2 mm gap in between. Eight square wave electric pulses (EP) of amplitude 120V (amplitude over a distance ratio of 600 V/cm), pulse duration of 5 ms and frequency of 1 Hz were generated by the electric pulse generator GT-01 (Faculty of Electrical Engineering, University of Ljubljana, Slovenia). After the electroporation, the cells were incubated for 5 min with 100 μl of FBS and then plated in an appropriate medium for further assays (flow cytometry and tube formation). Cell suspensions that were not exposed to EP served as control groups.

### Flow cytometry

The flow cytometry measurements of transfected cells with either of both plasmids encoding EGFP were performed in order to determine their tissue specificity. Two days after GET of pET-EGFP and pEGFP-N1 in different cell lines, cells were trypsinized, centrifuged for 5 min at 25°C at 1500 rpm, resuspended in 400 μl of phosphate buffered saline (PBS) and transferred to polystyrene round-bottom tubes (BD Biosciences, San Jose, CA, USA). The measurements of the percentage of transfected cells in each experimental group were performed with FACSCanto II flow cytometer; (BD Biosciences). For the excitation and detection of EGFP fluorescence a 488-nm laser (aircooled, 20 mW solid state) and 530/30-nm band-pass filter were used, respectively. To eliminate debris, 20000 cells were first gated, and afterward histogram of gated cells against their fluorescence intensity was recorded (software: BD FACSDiva V6.1.2). All of the experiments were repeated independently at least three times and each group consisted of three or more parallels.

Experimental groups *in vitro* were: addition of endotoxin-free water alone (control group; CTRL) or in combination with the application of electric pulses (EP group), addition of plasmid pET-EGFP (TS group) or pEGFP-N1 (CON group) alone or combined with the application of electric pulses (GET of TS plasmid; GET of CON plasmid).

### Tube formation assay

The tube formation was performed to determine the effect of endoglin silencing after CON and/or TS plasmids GET on the ability of mouse endothelial cells to form capillary like structures *in vitro*. Two days after transfection, 1.6 x 10^4^ 2H11 cells were plated on μ-Slide Angiogenesis (Ibidi, Munich, Germany) covered with BD Matrigel Basement Membrane Matrix, Phenol Red Free (BD Biosciences). Cells were incubated for 2 h until the formation of tubular complexes and stained with calcein AM (Sigma). Raw images were captured with a DP72 CCD camera (Olympus, Hamburg, Germany) connected to an IX-70 inverted microscope (Olympus) and converted into binary masks with AxioVision program (Carl Zeiss Microscopy GmbH, Jena, Germany). From binary masks, the total number of tubular complexes was determined by AngioQuant image analysis program [43]. The assay was performed independently three times and each group consisted of three or more parallels.

Experimental groups were: addition of endotoxin-free water alone (control group; CTRL) or in combination with the application of electric pulses (EP group), addition of plasmid pET-antiCD105 (TS group), pU6-antiCD105 (CON group) or pU6-SCR (SCR group) alone or combined with the application of electric pulses (GET of TS plasmid; GET of CON plasmid; GET of SCR plasmid).

### Ethics Statement

All animal experiments were conducted in accordance with the guidelines for animal experiments of the EU Directives and the permission obtained from the Ministry of Agriculture and the Environment of the Republic of Slovenia (Permission No. 34401-4/2012/2 which was given specifically for this study based on the approval of the National Ethics Committee for Experiments on Laboratory Animals, because Slovenia is a small country that does not have an Institutional Ethics Committee). The National Ethics Committee is under the auspices of the Ministry of Agriculture and Environment of the Republic of Slovenia. Animals were sacrificed by CO_2_ inhalation.

### Experimental animals

In the experiments, 6–8 week old female BALB/c mice (Harlan, Udine, Italy) were used. Mice were subjected to an adaptation period of 2 weeks before the experiments began. They were maintained in a 12 h light/dark cycle under specific pathogen-free conditions at a constant room temperature and humidity. Food and water were provided *ad libitum*. For induction of subcutaneous tumors, a suspension of 2 x 10^6^ TS/A cells, prepared from cell cultures *in vitro* in 0.1 ml of physiological solution, was injected into the right flank of mice.

Animals were randomly divided into experimental groups and subjected to a specific experimental protocol, when their tumors reached 3 mm in the largest diameter and were not vascularized or when their tumor volume reached 40 mm^3^ and had developed tumor blood vessels, respectively. With this division, we wanted to explore the vascular targeted efficacy of GET on tumors with different vascularization status and efficacy of TS plasmid in comparison with CON plasmid. When tumors reached 350 mm^3^, mice were sacrificed.

### In vivo GET

*In vivo* GET of plasmid into TS/A subcutaneous tumors was performed 3 times every second day (on days 0, 2 and 4) with the intratumoral injection of 12.5 μl (4 μg/μl) of plasmid (150 μg in total) in endotoxin-free H_2_O.

The experiments were performed independently two times, and each experimental group consisted of 6–11 mice per experiment. Experimental groups *in vivo* were: injection of endotoxin-free water alone (control group; CTRL) or in combination with the application of electric pulses (EP group), injection of plasmid pET-antiCD105 (TS group), pU6-antiCD105 (CON group) or pU6-SCR (SCR group) alone or combined with the application of electric pulses (GET of TS plasmid; GET of CON plasmid; GET of SCR plasmid).

Electric pulses generated by electroporator ELECTRO cell B10 HVLV (BETA tech, Saint-Orens-de-Gameville, France) were delivered 10 min after injection of plasmid DNA through two parallel stainless steel electrodes with 4 or 6 mm distance between them, depending on tumor size. Eight square-wave electric pulses of amplitude 240 V or 360 V (amplitude over distance ratio 600 V/cm), duration 5 ms at frequency 1 Hz, given in a perpendicular direction, were applied.

### Tumor growth

The therapeutic potential of GET *in vivo* was assessed by measuring the tumor size every second day after the therapy using digital Vernier caliper. Tumor volume was calculated according to the formula for ellipsoid: V = axbxc π/6, where a, b and c represent perpendicular tumor diameters [44]. From those, tumor growth curves were drawn as arithmetic means (AM) with bars representing standard errors (SEM). The weight of the mice was followed as a general index of systemic toxicity.

### Histology

Two days after the therapy, 3–4 mice from each experimental group were sacrificed and the tumors were excised. The tumors were fixed in IHC zinc fixative (BD Biosciences) and embedded in paraffin. Two consecutive 2-μm thick sections were cut from each paraffin block. The first section was used to estimate the percent of necrosis by 4 independent observers after staining with hematoxylin and eosin. The second section was used for immunohistochemical (IHC) staining of blood vessels. It was incubated with primary goat polyclonal antibodies against mouse CD105 (AF1320, R&D Systems, Minneapolis, MN, USA) at dilution 1:1800. As the colorogenic reagent, a peroxidase-conjugated streptavidin–biotin system (CTS008, Anti-Goat HRP-DAB Cell & Tissue Staining Kit, R&D Systems) was used and followed by hematoxylin counterstaining.

The IHC stained slides were observed under light microscopy and at least 5 images of viable tumor tissue from each slide were captured with a DP72 CCD camera (Olympus) connected to a BX-51 microscope (Olympus) under 40x magnification (numerical aperture 0.85). On the acquired images the number of CD105 positive blood vessels was determined.

### Statistical analysis

All data were tested for normality of distribution with the Shapiro-Wilk test. The differences between the experimental groups were statistically evaluated by one-way analysis of variance (one-way ANOVA) followed by a Holm-Sidak test for multiple comparison. A P-value of less than 0.05 was considered to be statistically significant. SigmaPlot Software (Systat Software, Chicago, IL, USA) was used for statistical analysis and graphical representation.

## Results

### Cell type specificity

**Fig 1:**
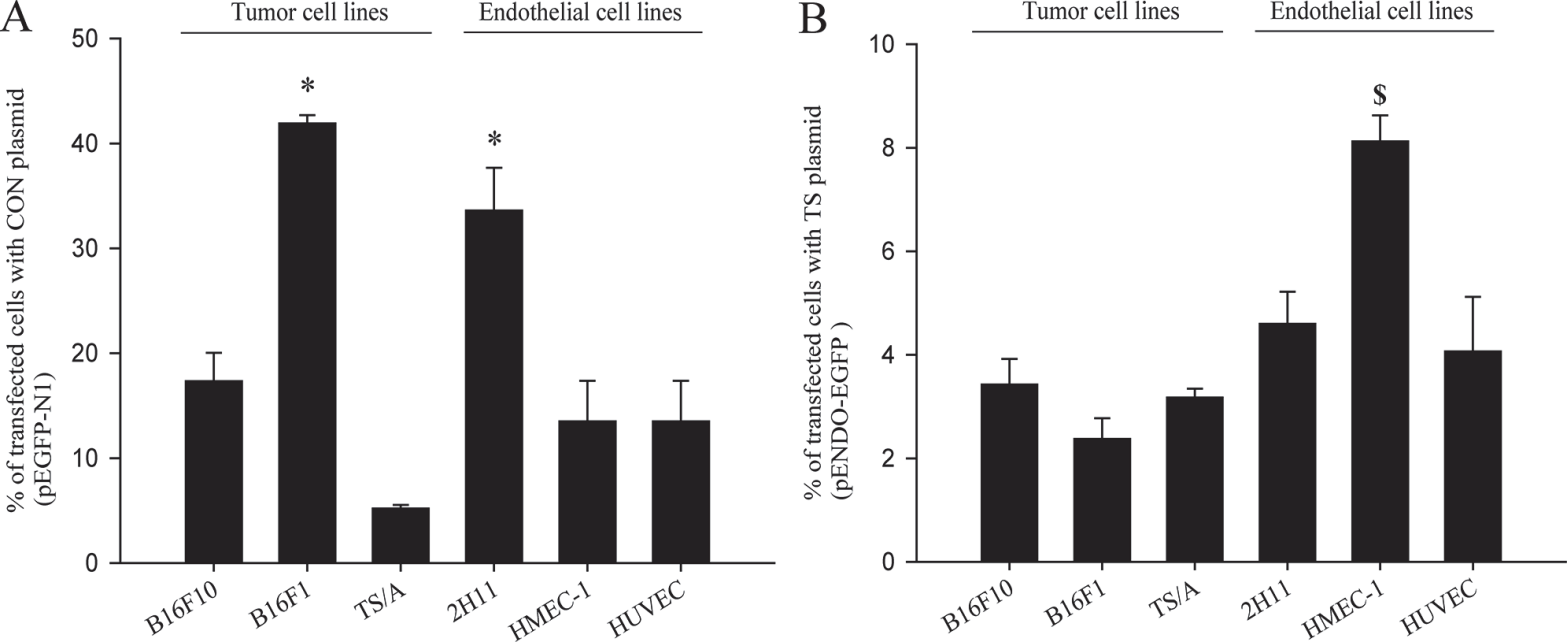
Transfection efficacy in tumor and endothelial cells *in vitro*. (A) Percent of transfected cells after gene electrotransfer (GET) of CON plasmid (pEGFP-N1) with statistically significant differences (*p<0.05) observed when comparing B16F1 and 2H11 to all of the other cell lines. (B) Percent of transfected cells after GET of TS plasmid (pET-EGFP) was statistically significant ($ p<0.05) when comparing HMEC-1 to all of the other cell lines. In general, higher transfection efficacy was observed with CON plasmid in comparison to TS plasmid. The results represent three independent experiments, n = 3 or more in each group in each experiment. The data represent AM ± SEM.

To determine tissue specificity of TS plasmid in different cell lines, the number of cells expressing EGFP was assessed by flow cytometry, after GET of plasmid encoding EGFP (pET-EGFP). In general, the transfection of tumor and endothelial cell lines was significantly higher and unspecific when using CON plasmid (pEGFP-N1) in comparison to TS plasmid. Using CON plasmid, no cell type specific transfection was observed; *i*.*e*. the highest was in B16F1 tumor cells, followed by 2H11, B16F10, HMEC-1, HUVEC, TS/A; endothelial and tumor cells (Fig 1A). These results indicate that transfection is cell type independent, since the statistically significant difference was observed between two different cell lines, B16F1 and 2H11.

On the other hand, when using TS plasmid, the pattern of predominantly higher transfection efficiency in endothelial cells was observed, indicating on its tissue specificity. Higher transfection efficacy in general was achieved in endothelial cell lines, compared to the other tumor cell types tested. Statistically significantly higher transfection efficacy was observed in HMEC-1 cell line, whereas the transfection was also high in two other endothelial cell lines, murine 2H11 and human HUVEC. Nearly two times lower transfection as observed in the HMEC-1 cell line, was obtained in non-endothelial, tumor cell lines; B16F10, TS/A and B16F1 (Fig 1B).

To sum up, the transfection efficacy when using CON plasmid was higher and unspecific, whereas when using TS plasmid the transfection efficacy was generally lower, but more pronounced in endothelial cell lines, indicating on its tissue specificity.

### Antiangiogenic effects *in vitro*

**Fig 2:**
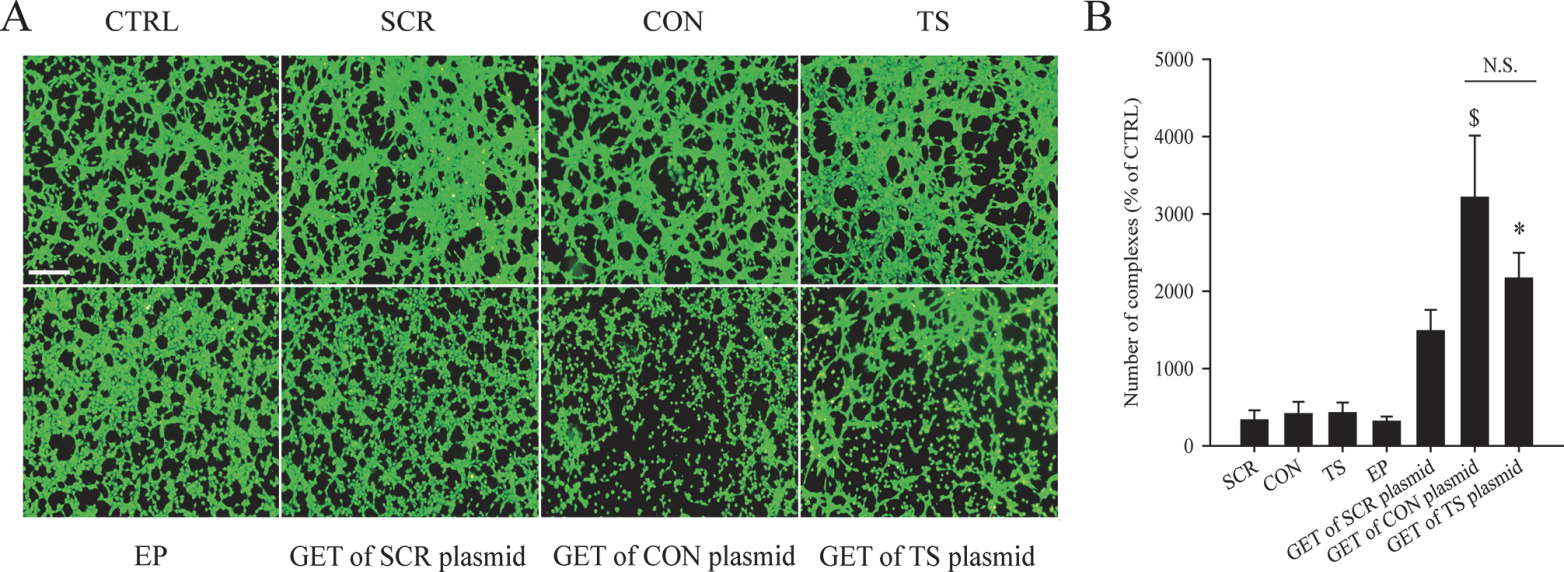
Endoglin silencing reduced tube formation of 2H11 endothelial cells *in vitro*. (A) Original images of tubular complexes in control and therapeutic groups; with addition of endotoxin-free water alone (control group; CTRL) or in combination with the application of electric pulses (EP group), addition of plasmid pET-antiCD105 (TS group), pU6-antiCD105 (CON group) or pU6-SCR (SCR group) alone or combined with the application of electric pulses (GET of TS plasmid; GET of CON plasmid; GET of SCR plasmid); scale bar = 400 μm. (B) The number of complexes normalized on the control group (CTRL); GET of CON plasmid ($ p<0.05) *vs*. all the other groups except GET of TS plasmid; GET of TS plasmid (* p<0.05) *vs*. CTRL, TS and EP group. The results represent three independent experiments, n = 3 or more in each group in each experiment for (B) and representative images for (A). The data represent AM ± SEM. N.S. represents statistically non-significant difference between the therapeutic groups.

The endothelial cell tube formation assay (*in vitro* angiogenesis assay) was performed with therapeutic plasmids silencing endoglin, one with constitutive (CON plasmid) and the other with the tissue specific promoter (TS plasmid). The antiangiogenic potential of GET of either of both plasmids was compared two days after GET. Results demonstrate that both therapies reduced formation of tubular complexes, indicating on the same antiangiogenic effect in mouse endothelial cell line 2H11 *in vitro* (Fig 2A), which in the pertinent control group was not observed. Furthermore, the analysis showed that the number of complexes, indicating disrupted tube formation, was moderate, but non-statistically elevated in group of GET of SCR plasmid, and highly, statistically significantly elevated in both of the therapeutic groups with no significant difference between them (Fig 2B). Based on the formation of tubular complexes in a tube formation assay, we can conclude that both therapeutic plasmids have antiangiogenic efficacy with similar activity.

### Antitumor efficacy

The murine mammary adenocarcinoma TS/A, growing in immunocompetent BALB/c mice, was used *in vivo* as tumor model where tumor cells do not express endoglin or ET, and therefore the effect of GET of plasmids silencing endoglin should be based on vascular targeted effects. Mice were divided into two groups with the only difference being the tumor volume at the time when the therapy was performed. The first group consisted of smaller tumors in avascular phase of growth (3–4 mm^3^; 3 mm in the longest diameter), while in the second group were bigger tumors in the exponential phase of growth (40 mm^3^; 5–6 mm in diameter) with developed vasculature. Adequate controls with the pertinent tumor volumes at the beginning of the experiments were included in both of the groups, and the therapy was performed on days 0, 2 and 4.

**Fig 3:**
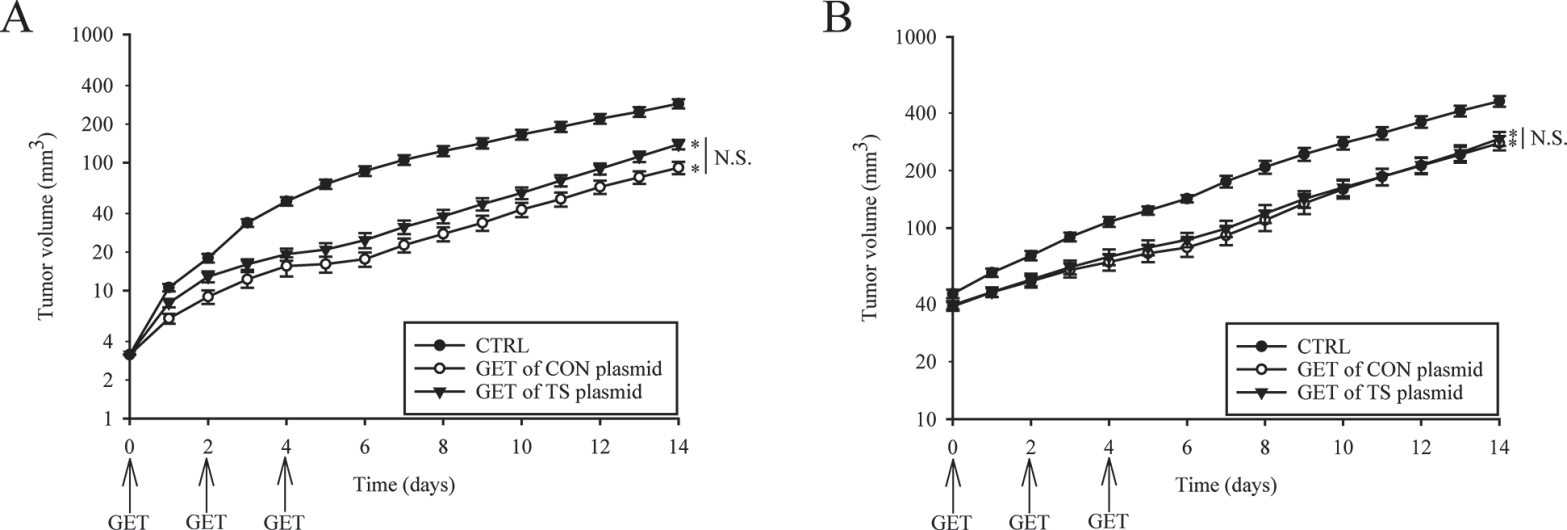
The tumor growth of smaller and bigger tumors was affected after the therapy. The growth of TS/A tumors exposed to intratumoral injection of endotoxin-free water alone (control group; CTRL) or therapeutic plasmids combined with the application of electric pulses (GET of TS plasmid; GET of CON plasmid), after triple GET of plasmids, was more affected in (A) smaller avascular tumors than in (B) bigger well vascularized tumors. The results represent two independent experiments, n = 6–11 mice for each experimental group in each experiment. The data represent AM ± SEM. N.S. represents statistically non-significant difference between the therapeutic groups and (*p<0.05) in both of the therapeutic groups (GET of CON and TS plasmid) *vs*. CTRL. Due to the clarity of the results, only main groups are shown.

Antitumor efficacy of GET of CON and TS plasmid silencing endoglin was pronounced during the course of the treatment and faded away after the treatment. The efficacy was in general more pronounced in treatment of smaller than in bigger tumors (Fig 3), and in both of the groups, the comparison of the efficacy of GET of CON and TS plasmid demonstrated non-significant therapeutic differences between them.

During the observation period, there were no changes of the animals’ weight, confirming the absence of systemic toxicity.

### Histological analysis

Histological analysis, with the determination of the extent of tumor necrosis and determination of the number of activated tumor blood vessels (CD105 positive cells), was performed to confirm the vascular targeted effects of GET of CON and TS plasmid in bigger, vascularized tumors.

**Fig 4:**
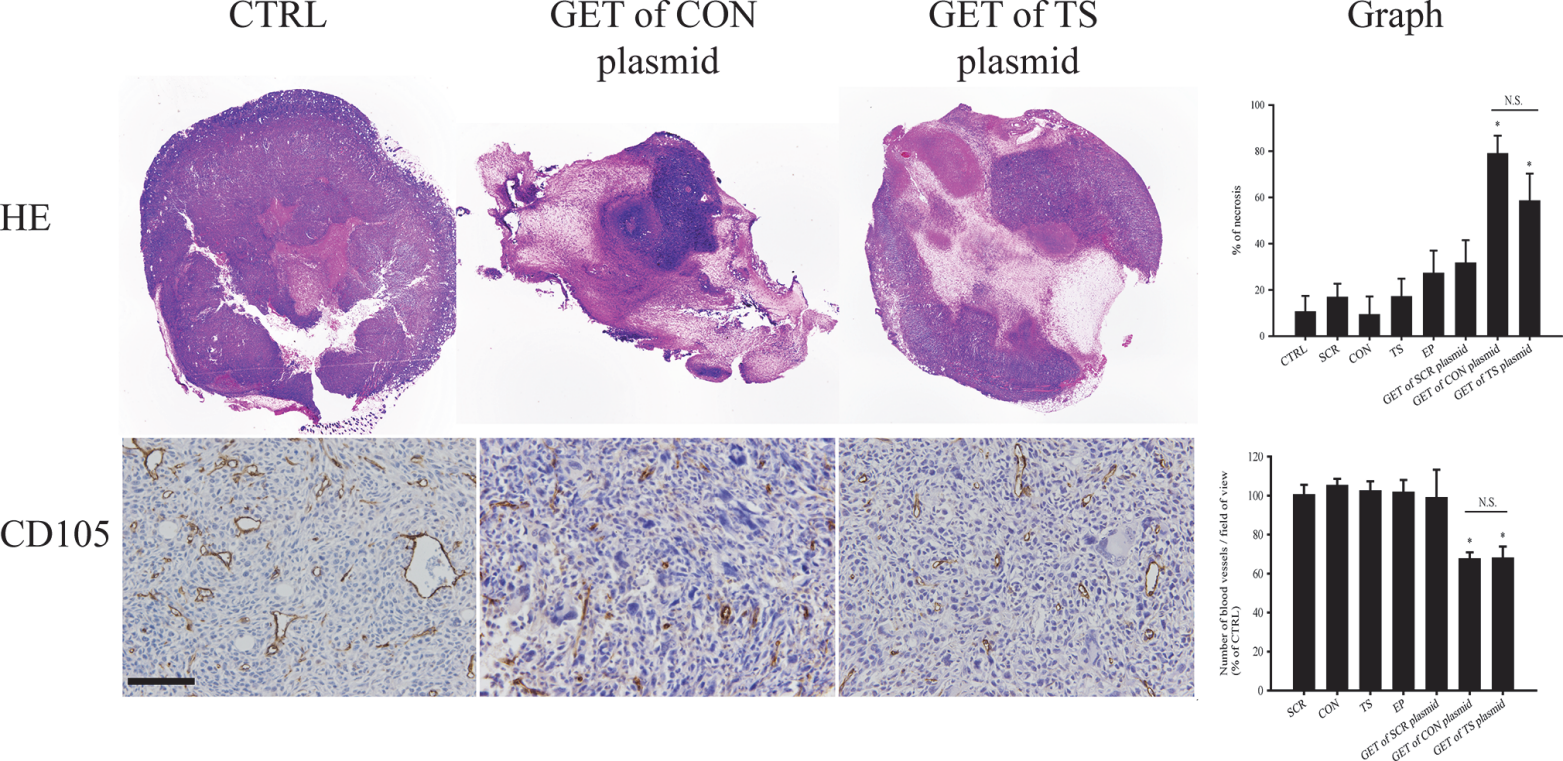
Increased tumor necrosis and reduced tumor vascularization after GET of therapeutic plasmids *in vivo*. Histologically stained and analyzed sections after treatments of TS/A tumors: intratumoral injection of endotoxin-free water alone (control group; CTRL) or in combination with the application of electric pulses (EP group), injection of plasmid pET-antiCD105 (TS group), pU6-antiCD105 (CON group) or pU6-SCR (SCR group) alone or combined with the application of electric pulses (GET of TS plasmid; GET of CON plasmid; GET of SCR plasmid). Triple GET of CON or TS plasmid increased necrosis (HE) and reduced the number of blood vessels (CD105) in TS/A tumors. The percentage of necrosis (upper graph) was statistically significantly increased (*p<0.05) in both of the therapeutic groups (GET of CON and TS plasmid) *vs*. all the pertinent control groups, with no difference between therapies, also seen in histological sections. The reduced number of blood vessels (CD105) was observed in histological sections of both therapeutic groups that were (lower graph) non-statistically significant to each other after analysis, although the reduction was statistically significant (*p<0.05) *vs*. all the pertinent control groups. The results represent one experiment, n = 3–4 mice for each experimental group and at least 5 analyzed fields of view for each mouse. The data represent AM ± SEM. N.S. represents statistically non-significant difference between the therapeutic groups. Scale bar = 100 μm.

The necrotic areas, observed 6 days after the beginning of treatments, were in general diffusely scattered throughout the tumor. The extent of tumor necrosis was statistically significantly increased in the group of tumors treated with GET of both therapeutic plasmids. In comparison to control group (CTRL), the necrosis has increased for 68.0 ± 8.8% in the therapeutic group using CON plasmid and for 47.9 ± 8.9% in therapeutic group using TS plasmid (Fig 4). The extent of the necrosis with GET of either plasmid was statistically significantly higher compared to all the pertinent control groups (CTRL, SCR, CON, TS, EP and GET of SCR plasmid). Again, there was no statistically significant difference in the efficacy between the therapeutic groups, with GET of the CON and TS plasmids (Fig 4).

In the remaining viable tumor areas, the number of stained activated blood vessels was statistically significantly reduced in both therapeutic groups (GET of CON and TS plasmid) in comparison to all the other pertinent groups for 32.8 ± 3.6% and 32.4 ± 6.13% when normalized on CTRL, respectively (Fig 4). Again, non-statistically significant difference in the effect between either of both therapeutic groups was observed.

## Discussion

GET of plasmids silencing endoglin, CON and TS plasmid, has good therapeutic potential for treatment of tumors *in vitro* and *in vivo*. First, we demonstrated that TS plasmid, the one with the tissue specific promoter of endothelial cell marker endothelin-1, is tissue specific and has almost the same antiangiogenic efficacy on tube formation as CON plasmid. Second, *in vivo*, we confirmed the good antitumor effect of GET of either of both plasmids in treatment of smaller tumors still in avascular phase of growth, as well as on bigger tumors, already well vascularized. In support to the observations on predominantly vascular targeted effects of endoglin, histological analysis has demonstrated an increase in necrosis and decrease in the number of blood vessels in therapeutic groups. A significant antitumor efficacy was observed in tumors in both therapeutic groups, in avascular and vascularized phase of growth, possibly due to both, the antiangiogenic and the vascular disrupting effect. Furthermore the study indicates on the potential use of TS plasmid in cancer gene therapy, which has the same efficacy as CON plasmid, and presumably displays the more favorable safety profile.

Therapeutic effects of monoclonal antibodies [19–21] targeting endoglin were already demonstrated, since endoglin expression is highly elevated during the tumor angiogenesis and vascular development, on the proliferating endothelial cells [45,46]. In the preclinical studies good antitumor effect was obtained [13], which led into the clinical trials on patients with advanced and refractory solid tumors. The antibody TRC105 [15] was applied systemically and when it was tested as a single agent, stable disease or better was achieved in 47% of patients. In combination with other treatments as VEGFR TKIs or Bevacizumab, 25% of patients had a reduction of tumor volume, though displaying some side effects such as headaches and telangiectasia [47].

Direct antitumor efficacy can be obtained by local GET of plasmids. After intratumoral plasmid injection, electroporation is used as a gene delivery system [48], which has proved its efficacy in several studies [49]. We have already demonstrated antitumor efficacy of this approach in murine tumor model, using CON plasmid, through vascular targeted effects [11]. A step further would be the use of GET of plasmids with tissue specific promoters [50] that would enable safer and more specific local gene therapy approach for targeting tumor vasculature. Some studies have already demonstrated specificity and efficacy of these plasmids with different endothelial cell markers. Danda *et al*. demonstrated that plasmid carrying the suicide gene was selectively expressed in the cancer cells (retinoblastoma) overexpressing Epithelial cell adhesion molecule (EpCAM) [51], and Dancer *et al*. demonstrated *in vivo* reduction of tumor volume and the number of blood vessels in the study targeting a suicide gene in the endothelium of transgenic mice [52]. However, the usefulness of these promoters was also demonstrated by several other studies [53–55].

Due to the limited number of studies targeting tumor vasculature with GET using plasmid with promoter specific for endothelial cells, in the first part of this study, we have evaluated the specificity of TS plasmid expressing EGFP (pET-EGFP) and demonstrated that it is a TS plasmid, which predominantly transfects endothelial cells. In comparison, pEGFP-N1, CON plasmid expressing EGFP, demonstrated unspecific and higher transfection efficacy, which is expected since it has viral constitutive promoter that is generally stronger and drives higher expression of the transgene soon after transfection [56]. The obtained results confirmed the results of our preliminary study, in which we had measured the transfection efficacy of both of the plasmids on a number of different cell lines [42], and are also in accordance with other results comparing the strength and transfection efficacy of different promoters in several cell lines [57]. In the present study, we extended the study to determine antiangiogenic effect of the two therapeutic plasmids silencing endoglin; CON and TS plasmid by tube formation assay. The GET of both of the plasmids used, hindered and prevented the proper tube formation, indicating their antiangiogenic potential. In accordance to our results, the same effect was also demonstrated in other studies using antisense oligonucleotides [58], anti-endoglin nanobodies [59], siRNAs [12] and CON plasmid [11]. The destruction of tube formation was also observed in the group of GET of SCR plasmid. Similar results were previously obtained in our [11] and other studies [60,61], indicating that plasmid backbone alone, thus devoid of therapeutic genes, can affect biological properties of the cells *in vitro* and *in vivo*. Our results *in vivo* provide encouraging evidence that endoglin is indeed important for angiogenesis and its silencing is effectively preventing it. To confirm this *in vitro* obtained evidence, an *in vivo* study on TS/A tumors in mice was conducted.

In order to overcome the limitations of too short-lived (< 5 min) siRNA molecules [62], plasmids encoding shRNA against endoglin were constructed (CON and TS). Studies have shown that the utilization of vector-based shRNA allows longer and more stable expression of shRNA, therefore a better therapeutic potential can be achieved [63–65]. The antitumor and vascular targeted effects of GET comparing the CON and TS plasmid were assessed on smaller tumors in avascular phase of growth, and on bigger well vascularized tumors. The vascular targeted efficacy was clearly demonstrated by slower tumor growth and prolongation of the avascular phase of tumor growth. In spite of this expectation, the efficacy of our therapy lasted only during the repetitive treatments and thereafter the effect was overruled by the tumor growth. Similar efficacy on smaller, avascular tumors was also demonstrated in other studies targeting endoglin using anti-endoglin mAb [20,21,66]. The reason for these results in our study could be in the fast growing TS/A tumors that do not express endoglin, therefore the therapy was affecting only tumor endothelial cells. Nevertheless, the effectiveness was more pronounced compared to our previous study where repetitive treatment with siRNA against endoglin was used. Tumor growth delay was ~ 6 days after GET of shRNA and ~ 3 days after GET of siRNA [12].

Therapy of tumors with either CON or TS plasmids was effective on smaller tumors, and to further demonstrate the vascular disrupting effect of endoglin silencing, with the same treatment protocol we tested the therapy on bigger, well vascularized tumors (40 mm^3^). The antitumor efficacy in bigger tumors was significant, however less pronounced compared to efficacy in smaller tumors. Efficacy in the established tumors was also observed in other studies [66,67] where mAb were used. Again, in our study, no difference in the efficacy was observed if GET was performed with CON or TS plasmid. We hypothesize that the antitumor efficacy on bigger tumors is due to dual vascular targeted effects, the antiangiogenic and vascular disrupting effect. In order to support this presumption, we performed the histology of the treated tumors. The percentage of necrosis in both of the therapeutic groups, in comparison to pertinent control groups, was highly elevated (68.0 ± 8.8% in GET of CON plasmid and 47.9 ± 8.9% in GET of TS plasmid) demonstrating that silencing of endoglin indeed affected the established tumor vessels, thus producing necrotic cell death. Antiangiogenic effect was obvious in the remaining peripheral viable parts of the tumors resulting in the reduced number of activated blood vessels. It was demonstrated that both of the therapies reduced their number for approximately 33% in comparison to the control group. Thus, the results of the present study directly support and extend the results of our previous study, where this dual action of endoglin silencing was proposed [11].

These results indicate that targeting endoglin is an attractive approach for specifically targeting tumor blood vessels and consequently affecting tumor growth. Furthermore, another step forward in comparison to previous studies was *in vitro* and *in vivo* testing of GET of TS plasmid silencing endoglin, with tissue specific polymerase II promoter for endothelin-1, marker for endothelial cells. The direct comparison between the results obtained with reporter gene (EGFP) and therapeutic gene (silencing endoglin) cannot be performed due to the difference in the use of constitutive promoters, CMV vs. U6. Although one would expect a better effect of GET of CON plasmid, due to the use of polymerase III U6 promoter that is robust and has constitutive activity across multiple cell types, this was not the case in our study. Indeed, we showed that GET of TS plasmid, which has endothelin-1 specific polymerase II promoter, can effectively provide expression of shRNA in specifically targeted cells and can therefore minimize unwanted expression in surrounding tissue, which is more desirable for use in the treatment of human diseases. Using GET of this plasmid we were able to demonstrate that it is specific, safe and has slightly lower, but non-statistically significant efficacy as the GET of CON plasmid. The comparable expression efficacy of shRNA molecules under the control of tissue specific (liver polymerase II) and constitutive (U6) promoter was also observed in study of Giering *et al*. [68], additionally confirming our results. Therefore, our approach can represent a novel alternative to other therapeutic approaches that aim at silencing endoglin, which represents also an alternative target in VEGF resistant (unresponsive) tumors.
